# Anti-VP6 VHH: An Experimental Treatment for Rotavirus A-Associated Disease

**DOI:** 10.1371/journal.pone.0162351

**Published:** 2016-09-07

**Authors:** Lucía Maffey, Celina G. Vega, Samuel Miño, Lorena Garaicoechea, Viviana Parreño

**Affiliations:** 1 Instituto de Virología, Centro de Investigaciones en Ciencias Veterinarias y Agronómicas, INTA Castelar, Buenos Aires, Argentina; 2 Consejo Nacional de Investigaciones Científicas y Tecnológicas, CONICET, Buenos Aires, Argentina; Instituto Butantan, BRAZIL

## Abstract

Species A Rotaviruses (RVA) remain a leading cause of mortality in children under 5 years of age. Current treatment options are limited. We assessed the efficacy of two VP6-specific llama-derived heavy chain antibody fragments (VHH) -2KD1 and 3B2- as an oral prophylactic and therapeutic treatment against RVA-induced diarrhea in a neonatal mouse model inoculated with virulent murine RVA (ECw, G16P[16]I7). Joint therapeutic administration of 2KD1+3B2 (200 μg/dose) successfully reduced diarrhea duration, RVA infection severity and virus shedding in feces. While the same dose of 2KD1 or 3B2 (200 μg) significantly reduced duration of RVA-induced diarrhea, 2KD1 was more effective in diminishing the severity of intestinal infection and RVA shedding in feces, perhaps because 2KD1 presented higher binding affinity for RVA particles than 3B2. Neither prophylactic nor therapeutic administration of the VHH interfered with the host’s humoral immune response against RVA. When 2KD1 (200 μg) was administered after diarrhea development, it also significantly reduced RVA intestinal infection and fecal shedding. Host antibody responses against the oral VHH treatment were not detected, nor did viral escape mutants. Our findings show that oral administration of anti-VP6 VHH constitute, not only an effective prophylactic treatment against RVA-associated diarrhea, but also a safe therapeutic tool against RVA infection, even once diarrhea is present. Anti-VP6 VHH could be used complementary to ongoing vaccination, especially in populations that have shown lower immunization efficacy. These VHH could also be scaled-up to develop pediatric medication or functional food like infant milk formulas that might help treat RVA diarrhea.

## Introduction

Species A rotaviruses (RVA) remain a leading cause of child mortality, causing around 215,000 deaths of <5 y old children in 2013, mainly in developing countries [1]. Ongoing clinical management of RVA-associated diarrhea is non-specific and mainly based on preventing the symptoms and dehydration through the administration of Oral Rehydration Salts (ORS), zinc supplementation and continued feeding [2]. Several non-specific therapies have been tested, including the use of different probiotics [3–5], drugs such as nitazoxanide [6], strict sanitation and hand hygiene—with limited benefits—[7] and herbal compounds used in folk medicine for the treatment of diarrhea [8,9]. Several attempts to develop a RVA-specific passive immune treatments have been made, including breast-feeding promotion [10], administration of RVA-specific bovine colostrum [11], egg yolk polyclonal IgY antibodies (Abs) to RVA [12–14] and RVA-specific monoclonal Abs [15,16]. Immune bovine colostrum was demonstrated to significantly reduce the risk of RVA gastroenteritis in children during an outbreak and the number of days with RVA-associated diarrhea [17]. On the other hand, IgY egg yolk immunoglobulins derived from hyperimmunized hens were shown to confer protection against RVA-associated diarrhea in gnotobiotic piglets [14] and a reduction of stool output in children spending short periods in hospitals [12,18,19]. However, none of these remedies is available as a commercial preparation, probably due to potentially adverse effects concerning the risk of allergic reactions and contamination with adventitious viruses [10,19]. Monoclonal Abs (mAbs) to RVA have been only tested prophylactically in the suckling mouse model for simian RVA (RRV strain) infection in which mouse pups were successfully protected against RVA infection and diarrhea when administered thirty minutes before infection [15,16]. However, up to now, this passive immune strategy has not been implemented as a treatment for RVA-associated diarrhea. While Abs can have high specificity, the doses required to treat patients are typically large [20]. Also, conventional mAbs face denaturation because of the stomach’s acidic pH and degradation by proteases in the stomach and intestine into small peptides or amino acids which are subsequently absorbed [21].

All camelid species possess a functional class of Abs devoid of light chains, referred to as heavy-chain Abs [22], whose single N-terminal variable domain (VHH or nanobody®) binds antigen without requiring domain pairing [23]. VHH consist of only one polypeptide chain and are expressed to a high level in microorganisms [24,25], mammalian cell lines, insect larvae [26] and plants [27,28] with low production costs [29], which represents an advantage for their application in massive treatment strategies. In the present work, we investigated llama-derived single-chain Ab fragments -VHH- directed to VP6 as a treatment against RVA-associated disease.

RVA have a genome consisting of 11 segments of double-stranded RNA that mainly encode a single polypeptide, allowing the virus to express six structural proteins (VPs) and five non-structural proteins (NSPs) [30]. RVA infections induce neutralizing Abs against outer capsid proteins VP4 and VP7 [31]. Upon primary infection these neutralizing Abs are mainly serotype specific and can hence protect against homosubtypic infections [32]. The inner capsid is composed of VP6 proteins which are vastly conserved, highly immunogenic and constitute the target antigen of most immunodiagnostic tests [33]. VP6 is not a target for conventional neutralizing Abs [34,35], although VP6-specific IgA mAbs were shown to induce intracellular viral inactivation during transcytosis as a mechanism of host defense against RVA infection in a “backpack model” in BALB/c mice [34]. Previous studies showed that VHH directed to VP6 are able to neutralize a wide range of RVA strains [36,37], suggesting that the conserved nature of this protein allows for cross-targeting of RVA strains.

VHH have a molecular weight of only 12–15 kDa rather than the 150 kDa of conventional Abs, and their more compact architecture allows them to access hidden targets [29] inaccessible to conventional Abs. They can resist temperatures above 90°C [38] and have a chemical stability of 60 kJ/mol−1 [39,40]. VHH are also resistant to other denaturing conditions, such as extreme pH [23,39] and high urea concentrations [41]. All these features encourage the use of VHH in oral therapies. VHH present high stability and therefore can be incubated for up to 200 hours at 37°C [42] or maintained for months at 4°C [29] without losing antigen-binding capacity. Particularly, lyophilized anti-RVA VHH together with rice water could be stored for a year at room temperature without losing their neutralizing activity against RVA [43].

Up to now, two independent VHH libraries to RVA have been generated from immunized llamas [44,45]. Van der Vaart and coworkers developed a library using the complete RVA virion from the simian RRV strain G3P[3]I2 (including clones ARP1 and ARP3) while Garaicoechea *et al*. used recombinant VP6 from bovine C486 strain G6P[1]I2 (including clones 2KD1 and 3B2) [37,44]. In both cases, VHH recognized RVA intermediate layer VP6 even though the exact epitopes have not yet been elucidated [37,45].

Some of these VHH also showed virus neutralization activity *in vivo* against different strains of RVA. In several previous studies, *Lactobacillus paracasei* that expressed anchored anti-RVA VHH clones ARP1 and/or ARP3 were constructed, aiming at obtaining a synergy between the effects of probiotics on diarrhea and the specific properties of ARP1/ARP3 against RVA [46–48]. ARP1 was also used to create an anti-RVA product by engineering rice plants to express the VHH (MucoRice-ARP1) which could be used as rice powder or rice water [43]. Both formulations achieved a significant reduction of diarrhea duration and disease severity in the neonatal mouse model for simian RRV infection when administered both before and shortly after viral inoculation [43,46–48]. More recently, ARP1 was tested in a clinical trial involving infants with RVA-associated diarrhea in Bangladesh where it successfully reduced the stool output but did not modify the duration of the diarrhea or the viral shedding [49].

Regarding VHH developed by our group, administration of clone 3B2 successfully prevented the occurrence of RVA-induced diarrhea in the gnotobiotic pig model for human RVA infection [36]. Both 2KD1 and 3B2 clones have been administered to neonatal mice before infection with murine RVA, and each of them conferred partial protection against diarrhea and significantly reduced virus shedding [37]. The therapeutic treatment of RVA-associated diarrhea using VHH Abs has never been systematically addressed. Moreover, little is known about the usefulness of VP6-specific VHH Abs as a treatment to RVA once virus infection has already induced diarrhea.

Here, we present the biological properties of VHH 2KD1 and 3B2 for prophylactic and post-infection therapeutic treatment of RVA in a neonatal mouse model with an extended time frame and sequential sampling that allowed us a more thorough exploration of how these VHH impair the development of RVA infection and disease. We also addressed concerns about the possible development of a host’s anti-VHH immune response upon treatment with VHH, a potential interference of the treatment with the host’s immune response against RVA, and the potential emergence of viral VP6 escape mutants due to the treatment.

## Materials and Methods

### Anti-VP6 VHH production

Anti-RVA VHH 2KD1 and 3B2 were obtained from an immune library of a llama immunized with recombinant inner capsid protein VP6 (from bovine RVA C486 strain G6P[1]I2), as previously described [37]. Both clones’ sequences are available at GenBank with the following accession numbers: JC36618 (2KD1), JC036616 (3B2). For this study, cDNA from both clones were re cloned using the restriction enzymes NcoI and NotI into the expression vector pHEN6. *Escherichia coli* XL-1 Blue were freshly transformed with the plasmid constructs. Both VHH clones were later purified from the periplasmic extract using a High-Trap HP Ni-chelating column (Amersham Biosciences) as previously described [37]. Purity of the VHH was assessed by Coomassie Blue staining of SDS-PAGE in which no significant contamination was observed (S1 Fig). Both VHH were then re suspended in commercial sterile saline solution (NaCl 0.9% p/v, pH 7.3) in a concentration of 3.9 mg/ml for 2KD1 and 7.29 mg/ml for 3B2, according to Nanodrop measures. These values were equivalent to an Ab titer of 32,768 (11.90 ng) and 65,536 (11.12 ng) respectively when tittered in an ELISA that measures VHH titers against RVA VP6, as described below in “Determination of VHH binding specificity” section, indicating that the molecules were expressed not only with good yield (mean value of 9.2 mg/L of culture) but also with optimal functionality.

### Virus

Neonatal BALB/c mice were infected with murine RVA strain ECw (G16P[16]I7) kindly provided by Dr. Alejandro Castello, Quilmes University with the permission of Ninguo Feng and Harry Greenberg. Mice were euthanized at Post Inoculation Day (PID) 4 and the intestinal contents were filtered and diluted in minimal essential medium (MEM; Invitrogen). The viral infectivity titer was determined by Cell Culture Immunofluorescence (CCIF) assay and expressed in Focus Forming Units (FFU)/ml). For viral inoculation of neonatal mice we used a volume of 100 μl, containing a total of 1778 times the dose that caused diarrhea in 50% of the suckling mice (DD_50_) equivalent to 10^3^ FFU. Murine ECw RVA attenuated by multiple cell culture passage was propagated in MA-104 cells (10^4^ FFU/ml) and used in Enzyme-Linked ImmunoSorbent Assays (ELISA) and CCIF assays.

### Animals

BALB/c mice were purchased from the breeding facilities at the Veterinary College, La Plata University and mated to produce offspring under regulated conditions. Pregnant dams were housed individually and normal pellet diet and water were provided *ad libitum*. In order to improve the housing conditions of the dams and their litters, we provided cotton balls for nesting purposes and sunflower seeds to enrich the protein content of the normal pellet diet. Neonatal mice were euthanized by manual beheading and adult mice by cervical dislocation after mild sedation with Isoflurane (inhalation for 30 seconds). Prior to the experiments, a protocol was established for early/humane endpoints for animals that presented severe illness before the experimental endpoint. Mice pups were euthanized if they presented severe lesions caused by improper intragastrical administration of the treatment or if they showed signs of severe dehydration, generally caused by a combination of the RVA-induced diarrhea and deficient lactation. Efforts were made at all stages of the experiments to minimize the suffering of animals. The local Institutional Committee for the Care and Use of Experimental Animals approved the followed protocol (Comité Institucional para el cuidado y uso de animales de experimentación–CICUAE- Protocol number 26/2014, CICVyA, INTA-Castelar, Argentina).

### Experimental design

**Fig 1:**
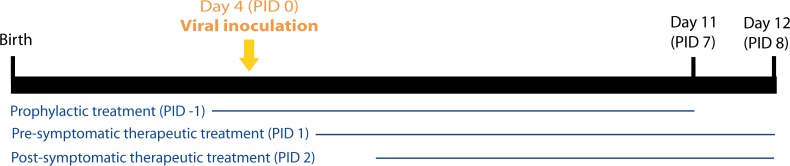
Experimental design. The thin horizontal lines represent the duration of the treatment with VHH. On PID 0, all groups were inoculated with 1778 DD_50_ of murine RVA (ECw). Prophylactic groups received the first daily doses of VHH on PID -1 and on PID 0, two hours before they were challenged with the viral inoculum. Pre-symptomatic and post-symptomatic treatments began on PID 1 and PID 2. Final euthanasia was performed at PID 7 for prophylactic groups and at PID 8 for therapeutic pre- and post-symptomatic groups. PID: Post Inoculation Day.

In all cases, four-day old pups were used for the experiments. Challenge conditions were previously standardized to produce diarrhea in 100% of untreated mice. For viral inoculation, pups received 5 μl of Sodium Bicarbonate (5% w/v), followed by 100 μl of murine ECw RVA (1778 DD_50_) at PID 0. Viral inoculation and all treatments were administered using a flexible intragastric gauge. All pups were examined daily to assess the occurrence of diarrhea by gentle abdominal palpation and collection of feces, by a researcher working blinded to the treatment assignment. Each litter was considered to have diarrhea if at least one pup from the litter presented soft or aqueous feces. In the present study, we modified previous schedules to analyze the development of the viral infection at the intestinal tissue and to monitor the evolvement of the disease until the resolution of the symptoms. In brief, control and treated mice were euthanized sequentially from PID 0 to 6 for prophylactic groups and from PID 0 to 7 for pre-symptomatic and post-symptomatic therapeutic groups (Fig 1). For prophylactic studies, final euthanasia was performed at PID 7 whereas for therapeutic groups it was conducted at PID 8 (Fig 1). After sacrifice, sera and intestinal samples were collected. Fecal and intestinal samples were tested by ELISA to determine viral shedding and intestinal samples were also analyzed by CCIF to detect infectious RVA particles.

#### Prophylactic assays

Both VHH, 2KD1 and 3B2, have been tested previously as prophylactics against RVA-associated diarrhea in the neonatal mouse model [26,37]; however, on those occasions all mice were euthanized at PID 4 and the only dosage used was 100 μg of VHH. We conducted new tests with a different time frame (Fig 1) and exploring the efficacy of two higher doses, using four litters of suckling mice for each daily treatment: 160 μg of 2KD1+3B2 (N = 31) and 200 μg of 2KD1+3B2 (N = 30). In both cases we used equal amounts of each VHH clone per dose and the treatment started on PID -1 and was administered until PID 6 (Fig 1). On PID 0, the VHH were administered 2 hours prior to viral challenge.

#### Pre-symptomatic therapeutic assays

Previous studies comprising different therapeutic treatments of RVA-induced diarrhea in mice started treatment administration between 2 and 24 hours post-infection [5,43,50]. For the therapeutic approach, we first conducted a pilot assay consisting of a dose/response test where we assessed three doses of treatment: 100 μg of 2KD1+3B2 (50 μg of each), 160 μg of 2KD1+3B2 (80 μg of each), 200 μg of 2KD1+3B2 (100 μg of each). Given that the lowest dosage (100 μg) did not modify the course of diarrhea and viral shedding in any way with respect to the untreated control (data not shown), this group was excluded from further analysis. At PID 0, all mice were inoculated with 1778 DD_50_ of murine ECw RVA (Fig 1). In order to study the therapeutic effect of 2KD1 and 3B2 against RVA-induced diarrhea, we started the treatment at PID 1 so that one cycle of viral replication *in vivo* was completed. This approach sought to avoid neutralization of the viral inoculum by VHH before successful infection was established. From PID 1 to 7, we tested four VHH daily treatments employing four litters of suckling mice for each: 160 μg of 2KD1+3B2 (80 μg of each) (N = 25), 200 μg of 2KD1+3B2 (100 μg of each) (N = 28), 200 μg of 3B2 (N = 24), 200 μg of 2KD1 (N = 30). Controls included: untreated ECw RVA-inoculated mice (untreated control group); untreated and not RVA-inoculated mice (normal control group). Anti-RVA murine Abs were determined by an isotype-specific ELISA in sera and intestinal samples at PID 0, 7 and 40. The occurrence of an immune response against the VHH was determined by measuring the presence of anti-VHH Abs titers at PID 0, 7 and 40 using a murine isotype-specific ELISA, and sera and intestinal samples of the treated mice as well as the control mice were tested. Translocation of VHH from the intestinal lumen to the serum of mice was determined up to PID 7 by an ELISA measuring functional levels of VHH.

#### Post-symptomatic therapeutic assays

Previous results from the standardization of the neonatal mouse model showed that mice infected with RVA present the first diarrheal symptoms at PID 2 (data not shown). If anti-RVA VHH were used to treat pediatric patients suffering RVA-associated diarrhea, the administration of the VHH would start after the onset of the first symptoms. In order to determine if post-symptomatic therapeutic treatment (starting at diarrhea onset, PID 2) with the VHH would achieve similar levels of efficacy than pre-symptomatic treatment (starting at PID 1), an additional group of mice, comprising four litters of suckling mice (N = 24), was added. Mouse pups were inoculated at PID 0 with 1778 DD_50_ of murine ECw RVA as described and received a daily dose of 200 μg of 2KD1 from PID 2 (Fig 1), at which time all mice in the group had already developed diarrhea, to PID 7. Normal control group and untreated control group were included.

### RVA antigen and virus detection

RVA shedding was detected in feces using an antigen capture ELISA as described previously [51,52]. Infectious virus titer was assessed by CCIF assay as detailed in previous studies [53]. We used an anti-RVA bovine IgG Ab labeled with FITC and both 2KD1 and 3B2 labeled with Alexa Fluor (Invitrogen) to reveal RVA-infected cells. Fluorescent cells were counted using a fluorescence microscope and titers were expressed as the reciprocal of the highest dilution showing RVA-infected cells.

### Murine isotype-specific Ab to ECw RVA ELISA

Anti-RVA IgA and IgG Ab titers were quantified in intestinal contents and sera, respectively. We decided to detect only the main isotype expected to appear in each kind of tissue, because of the small amount of sample available. We used an indirect ELISA based on the protocol described previously [52,54,55] with several modifications. Briefly, 96-well plates (NUNC-Maxisorp) were coated at 37°C with a pig hyperimmune serum against RVA and blocked with 10% non-fat milk (prepared in PBS-tween 0.5%). Murine ECw RVA (10^3^ FFU/ml) or mock-infected MA-104 cell supernatant fluids were added for another hour at 37°C. Serial 4-fold dilutions starting at 1:4 of each sample were assayed, followed by incubation with commercial Horseradish Peroxidase (HPR) labeled goat polyclonal Ab to murine IgA (1:3000) (KPL) or HRP-labeled goat polyclonal Abs to murine IgG (1:3000) (KPL) for 1 h at 37°C. Commercial hydrogen peroxide and ABTS (Sigma-Aldrich) were used as substrate/chromogen system, and the reaction was stopped with Sodium Dodecyl Sulfate (SDS) 5%. Serum and intestinal contents from two adult mice previously immunized with murine RVA were used as positive controls whereas serum and intestinal contents obtained from naïve mice served as negative controls for this assay. Optical density measured in mock-coated wells was subtracted from the one obtained in virus-coated wells and the cut off point for the assay was established as the mean plus three standard deviations of the corrected optical density measured in virus-coated PBS wells (blank).

### Host humoral immune response against VHH

Anti-VHH IgG and IgA Abs titers were quantified by ELISA in sera and intestinal samples respectively, of treated mice. We decided to detect only each isotype in the tissue where it represented the main Ab response, given the small amount of biological samples available. Briefly, 96 well plates (NUNC-Maxisorp) were coated with 50 ng per well of purified monovalent VHH 2KD1 or 3B2 and then blocked with 10% non-fat milk (in PBS-tween 0.5%). Serial four-fold dilutions of the samples starting at 1:4 were incubated for one h at 37°C followed by incubation with commercial HRP-labeled goat polyclonal Abs to murine IgG at a 1:3000 dilution (KPL) or commercial HPR-labeled goat polyclonal Ab to murine IgA at 1:3000 dilution (KPL). Commercial hydrogen peroxide and ABTS (Sigma-Aldrich) were used as substrate/chromogen system. The cut off point for the assay was established as the mean plus three standard deviations of the optical density measured in virus-coated PBS wells (blank). This ELISA was standardized using mice hyperimmune serum against VHH as positive control and serum samples from non-immunized mice as negative controls obtained from the experiment described below.

In order to study the immunogenicity of anti-RVA VHH when these were administered systemically, we performed an additional assay using adult mice. In all cases, mice were immunized subcutaneously twice with 50 ng of either 2KD1 or 3B2 at PID 0 and 21. Four groups, each comprising four adult mice, were used and received the following treatments: 2KD1 (50 ng); 2KD1 (50 ng) + adjuvants; 3B2 (50 ng); 3B2 (50 ng) + adjuvants. The employed adjuvants were Freund’s complete adjuvant for the first immunization and Freund’s incomplete adjuvant for the second one. Blood samples were obtained at PID 30 and 60 and later analyzed for seric anti-VHH IgG Abs.

### Screening of anti-VP6 VHH murine RVA escape mutants in mice samples

We analyzed intestinal and fecal samples from mice treated therapeutically with anti-VP6 VHH that were positive for RVA by CCIF or ELISA after 4–6 days of passive treatment administration. The main goal of this assay was to explore whether these samples contained RVA VP6 escape mutants, which may had emerged due to the passive treatment. We used viral inocula and samples from untreated mice as controls. The viral RNA was extracted using the QIAamp Viral RNA Mini Kit (Qiagen) following manufacturer’s instructions from a 200 μl starting volume of the centrifuged suspensions. The recovered RNA was eluted in 40 μl of elution buffer and stored at -80°C until further use. The whole encoding gene for VP6 (1356 bp) was amplified by RT-PCR as described previously [56]. The PCR amplicons were purified using ExoSAP-IT (Affymetrix USB), and sequenced for both sense/antisense using the dideoxynucleotide chain termination method with the ABI PRISM1 BigDye Terminator Cycle Sequencing Reaction kit (Perkin-Elmer Applied Biosystems) on an automated sequencer (ABI PRISMTM 3130) from the Sequencing Service, Biotechnology Institute (INTA, Argentina). Sequences were edited using BioEdit 7.0.9.0 Sequence Alignment Editor [57]. Comparisons between nucleotide and amino acidic sequences were conducted using MEGA5 [58]. A phylogenetic tree was constructed using the Maximum Likelihood method based on the Tamura 3-parameter model using a bootstrap statistical support of 1000 repetitions. Other reference murine RVA strains were obtained from GenBank.

### Proteolytic resistance of anti-VP6 VHH

In order to study the stability and protease resistance of the VHH we conducted two separated experiments in which we examined stability at low pH and resistance to gastric and intestinal enzymes for 2KD1 and 3B2, respectively. To study stability and resistance of the VHH in conditions that emulate the host’s stomach, aliquots consisting of 400 μg/ml of 2KD1 or 3B2 were incubated in Simulated Gastric Fluid (SGF; pH 1.2) with and without enzymes for up to one hour at 37°C. We also tested the resistance to gastric enzymes in the presence of ORS. The experiment included the following samples: 3B2+SGF (pH 1.2); 3B2+SGF without enzymes (pH 1.2); 3B2+SGF+ORS (pH 7.1). The SGF was prepared following the US Pharmacopeia guidelines, with a final porcine pepsin concentration of 0.32% w/v and pH 1.2. On the other hand, we examined the VHH’s resistance to intestinal proteases, by incubating 400 μg/ml of 2KD1 or 3B2 in Simulated Intestinal Fluid (SIF) with or without ORS for up to one hour at 37°C: 2KD1+SIF (pH 6.8); 2KD1+SIF without enzymes (pH 6.8); 2KD1+SIF+ORS (pH 7.1). The SIF was also prepared following the US Pharmacopeia guidelines, with a final pancreatine concentration of 1% w/v and pH 6.8. All samples were analyzed by an ELISA that measures VHH titers against RVA VP6 to determine the remaining percentage of functional VHH, as was previously described.

### Binding affinity and neutralization activity of 2KD1 and 3B2 against RVA

Binding affinity of each VHH clone was studied in a specific ELISA that detects VHH molecules attached to RVA particles. Briefly, 96-well plates (NUNC-Maxisorp) were coated at 37°C with a specific bovine IgG Ab against RVA (1:5000) and blocked with 10% non-fat milk (prepared in PBS-tween 0.5%). Bovine RVA (UK strain G6P[5]; 10^3^ FFU/ml) or mock-infected MA-104 cell supernatant fluids were added for another hour at 37°C. Serial five-fold dilutions were assayed for both clones starting at an initial concentration of 1.78 μg/ml for 3B2 and 7.6 μg/ml for 2KD1. This step was followed by incubation with rabbit hyperimmune serum against VHH and later with commercial Horseradish Peroxidase (HPR) labeled goat polyclonal Ab to rabbit IgG (1:2000; KPL) for 1 h at 37°C. Commercial hydrogen peroxide and ABTS (Sigma-Aldrich) were used as substrate/chromogen system, and the reaction was stopped with SDS 5%. We used 3B2 and 2KD1 VHH previously produced and characterized as positive controls whereas a non-related VHH was used as negative control for the assay. Optical density measured in mock-coated wells was subtracted from the one obtained in virus-coated wells and corrected ODs (at 405 nm) were plotted versus logarithm of the Abs concentration (μg/mL) and fitted using a sigmoidal dose-response analyses of non-linear data using GraphPad Prism 7 software. The EC50 (50% effective concentration) values were calculated and used for quantification and characterization of VHH binding affinity to RVA particles.

In order to provide further evidence regarding anti-RVA neutralization (VN) activity of the VHH, we conducted a fluorescent focus neutralization (FFN) test as previously described [52]. Given that 3B2 had already been tested by Vega *et al*. [36] for various human RVA strains, we determined VN titers of 2KD1 for the same strains. Also, we analyzed VN titers of both clones against the murine RVA strain used in mice experiments (ECw). Briefly, a four-fold dilution of each VHH clone (3B2, 2KD1 or a non-related clone) was mixed with an equal volume of RVA containing 100 DICT. The VN titer was expressed as the reciprocal of the highest sample dilution that resulted in 80% reduction in the number of fluorescent foci. The 80% reduction criterion was selected in order to be more stringent than in protocols considering only 50% reduction.

### Statistical analysis

Viral and Abs titers were log10 transformed and negative samples were assigned an arbitrary titer of 2 (log10 = 0.31) for calculations. The Area Under the Curve (AUC) was calculated for intestinal samples and feces. A one-way fixed effects ANOVA model (parametric) was used to compare duration of diarrhea and cumulative titers of virus shed in feces and intestines (Area Under the Curve -AUC- comparison) between treatments and with the untreated control when previous assumptions of normality and heterogeneity of variances were fulfilled using Shapiro-Wilks and Levene tests. Kruskal-Wallis rank sum test (non-parametric) was used when parametric tests’ assumptions were not complied. Proportions of mice with diarrhea for each day were compared using Fisher’s Exact Test. Differences in anti-RVA Ab titers among groups were evaluated by comparison of means at three different time-points post virus inoculation (PID 0, 8, 40) using a One-way ANOVA followed by Bonferroni pairwise comparison. Statistical significance was assessed at p = 0.05 for all comparisons. Statistical analyses were conducted using Infostat® statistical software.

## Results

### Prophylactic administration of anti-VP6 VHH significantly reduced diarrhea duration, intestinal RVA infection and RVA shedding in feces

**Fig 2:**
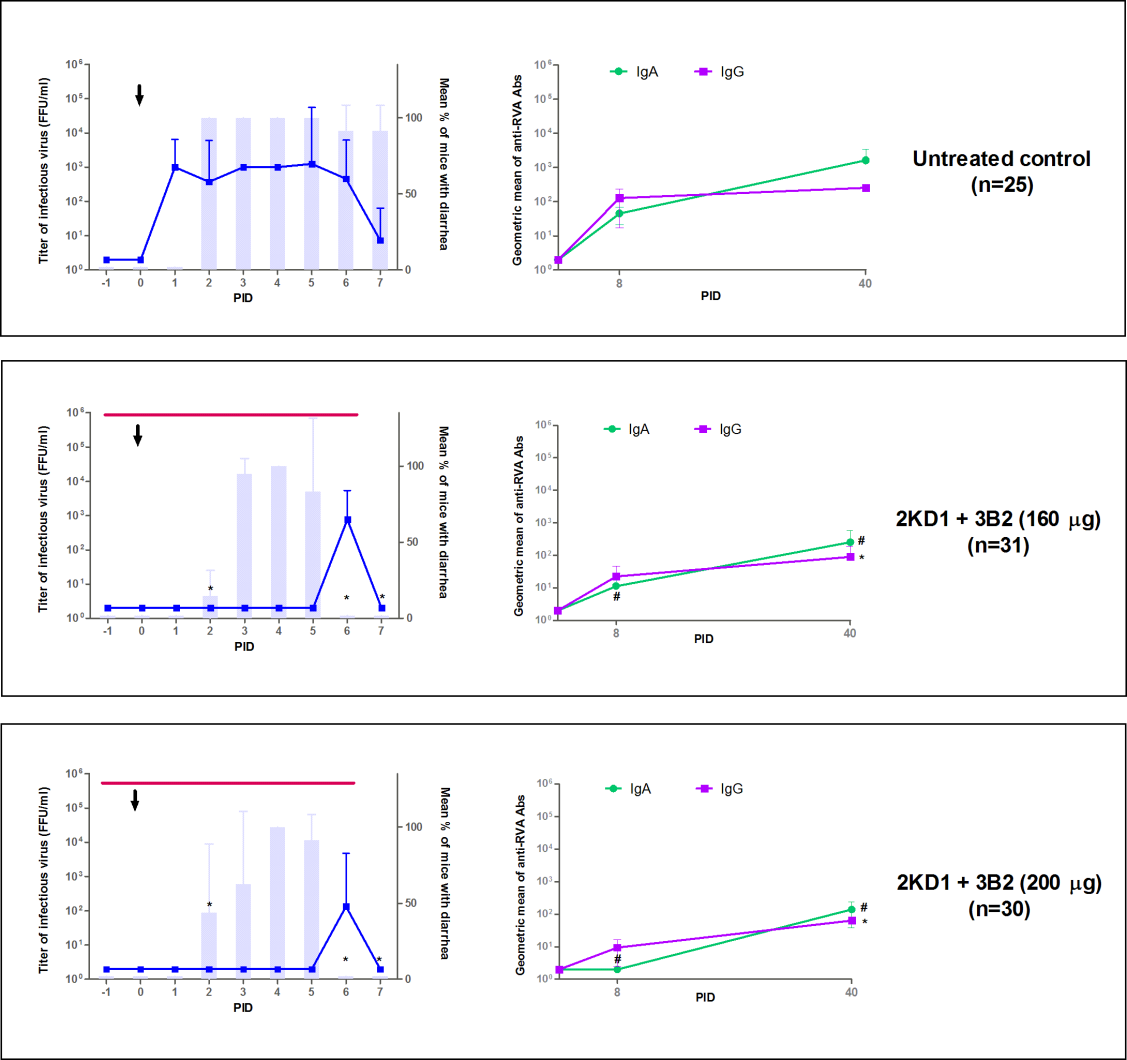
Effect of prophylactic administration of 2KD1+3B2 VHH on RVA-induced diarrhea and host humoral immune response against RVA. All mice were orally inoculated with 1778 DD_50_ of ECw RVA at day four of life (PID 0), as indicated by the arrow. Mice were euthanized sequentially to obtain intestinal tissue (3–4 mice per time point). Graphics on the left side of the panels depict intestinal infectious RVA titers (by CCIF) and diarrhea prevalence. Blue lines show the development of intestinal RVA disease whereas vertical bars show the mean percentage of mice with diarrhea per group. The * symbol indicates that the percentage of mice with diarrhea was significantly lower than in the untreated control group (Fisher Exact test). The thin horizontal line indicates the duration of VHH passive treatment. Graphics on the right side of the panel display humoral immune responses against RVA. Anti-RVA IgA in intestinal samples and serum IgG Ab titers were determined by ELISA at PIDs 0, 8 and 40. The # and * symbols indicate that the Ab titers of intestinal IgA and serum IgG, respectively, were significantly lower than the corresponding Ab titers in the untreated control group (One-way ANOVA). PID: Post Inoculation day.

The aim of the present experiment was to evaluate the protection of combined orally administered VP6-specific VHH (clones 3B2 and 2KD1) against murine RVA-associated diarrhea in the neonatal mouse model (Fig 1). At PID 0, all mice were infected with 1778 DD_50_ of murine ECw RVA (G16P[16]I7) two hours after VHH treatment. Fig 2 shows the presence of diarrhea and infectious RVA particles in the intestines of all groups, except for non-inoculated control mice that did not present viral titers at any time (data not shown). Prophylactic administration of 2KD1+3B2 VHH significantly delayed the onset of diarrhea, reducing the percentage of affected mice at PID 2 from 100% in the untreated group to 15% in 160 μg VHH mix/dose (p<0.0001) and 44% in the 200 μg VHH mix/dose (p<0.0001) (Fig 2). At this experimental time, the percentage of mice showing diarrhea was significantly higher in 200 μg VHH group than in the 160 μg VHH one (p = 0.02). However, no further differences were observed from PID 3 on. Furthermore, diarrhea duration was reduced in all VHH-treated mice to 3.25 days (160 μg 2KD1+3B2) and 3.5 days (200 μg 2KD1+3B2) compared to 6.75 days in the untreated control (p = 0.001).

**Fig 3:**
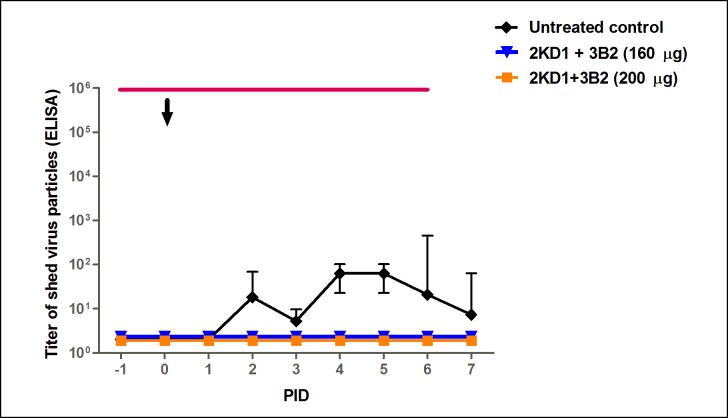
Effect of prophylactic administration of 2KD1+3B2 VHH on RVA shedding in feces. Mean titer of virus shed (determined by ELISA) detected daily in pooled feces for mice treated prophylactically with VHH and the untreated control group. Treated groups did not display detectable fecal shedding at any time. The arrow shows inoculation time (PID 0) and the thin horizontal line indicates the duration of VHH passive treatment. PID: Post Inoculation Day.

Viral load in the intestinal tissue was also assessed by CCIF assay using samples from sequential euthanasia (Fig 2). None of the VHH-treated groups displayed detectable RVA infectious particles in intestinal samples from PID 0 to 5, whereas mice in the untreated group control showed persisting viral titers from PID 1 on until the end of the experiment. On the whole, this accounted for a significant reduction in the severity of intestinal infection between groups receiving the VHH passive treatment and the untreated control (AUC comparison, p<0.0001). At PID 6, a single peak was observed in 75% (3/4) of the litters from both groups receiving the passive treatment (Fig 2), which implied no significant difference between them (p>0.05). No virus shedding was detected by ELISA in the feces from treated mice whereas the untreated control experienced persisting fecal shedding from PID 2 to 7 (Fig 3).

In order to study whether administration of VHH could modify the Ab responses against RVA, murine anti-RVA IgG and IgA Abs were evaluated in serum samples and intestinal contents respectively at PID 0, 8 and 40 (Fig 2). All animals from all experimental groups developed immune responses to RVA during the experiment. At PID 8, mice treated with the highest dose of VHH (200 μg) showed no detectable anti-RVA IgA titers in intestinal tissue, in contrast with animals treated with 160 μg VHH and the untreated control group. No significant differences were found between the latter two groups (p>0.05) (Fig 2). From PID 8 to 40, anti-RVA IgA Ab titers rose in all experimental groups, even for animals in 200 μg VHH group. However, at PID 40 the untreated control group showed an average IgA Ab titer of 1625 whereas mice receiving 160 μg and 200 μg presented average IgA Ab titers of 256 and 141 respectively, which implied a significant reduction when compared to the control (p = 0,001) (Fig 2). Serum anti-RVA IgG Abs levels showed a similar pattern. At PID 8, mice treated prophylactically with 200 μg of VHH showed one-fold lower Ab titers in serum samples than the untreated mice (p = 0.006) with an average Ab titer of 9.5 versus 128 in the control group. On the other hand, mice receiving 160 μg of the passive treatment presented an average IgG Ab titer of 22, similarly to the results obtained in the control group (p>0.05). At PID 40, both treated groups showed statistically significant differences with untreated mice (p = 0.002), even when IgG Ab titers in all groups of animals were higher at this experimental time point than in all the previous ones: an average IgG Ab titer of 90 and 64 in mice treated with 160 μg and 200 μg of 2KD1+3B2 respectively.

### Pre-symptomatic therapeutic administration of anti-VP6 VHH reduced duration and severity of diarrhea and RVA shedding in feces

**Fig 4:**
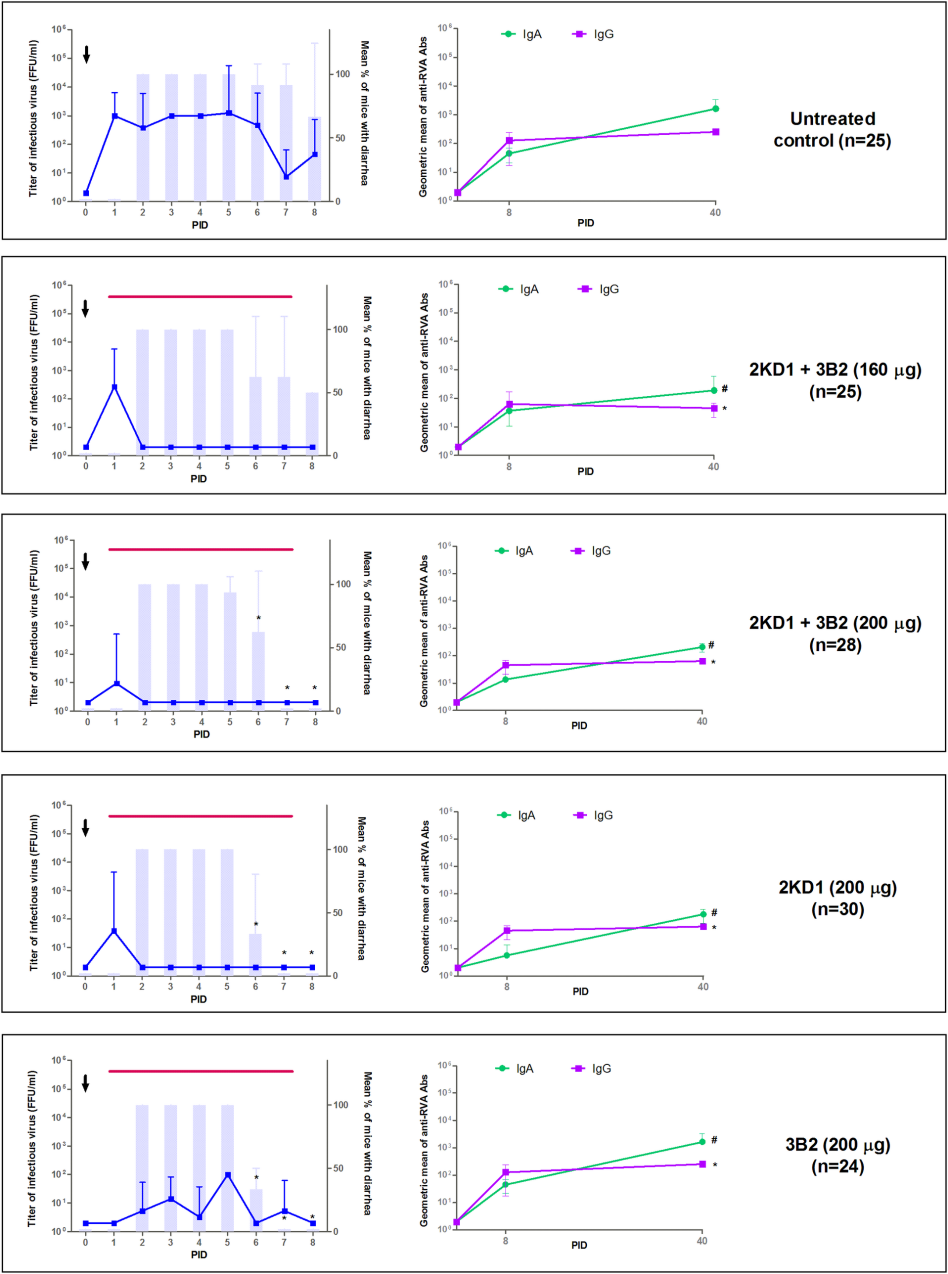
Effect of pre-symptomatic therapeutic administration of VHH on RVA-induced diarrhea and host humoral immune response against RVA. All mice were orally inoculated with ECw RVA (1778 DD_50_) at day four of life (PID 0), as indicated by the arrow. Mice were euthanized sequentially to obtain intestinal tissue (3–4 mice per time point). Graphics on the left side of the panel depict intestinal infectious RVA titer and the incidence of diarrhea. Blue lines show the development of intestinal RVA disease whereas vertical bars show the mean percentage of mice with diarrhea per group. The * symbol indicates that the percentage of mice with diarrhea was significantly lower than in the untreated control group (Fisher Exact test). The thin horizontal purple line indicates the duration of VHH passive treatment. Graphics on the right side of the panel display mice humoral immune response against RVA. Anti-RVA IgA in intestinal samples and serum IgG Ab titers were determined by ELISA at PID 0, 8 and 40. The # and * symbols indicate that Ab titers of intestinal IgA and serum IgG were significantly lower than Ab titers in the untreated control group (One-way ANOVA). PID: Post Inoculation Day.

Four day-old mouse pups were inoculated with murine ECw RVA strain (G16P[16]I7)–PID 0-. From PID 1 (24 h post-virus inoculation) to PID 7 included (see Fig 1), four groups of mice received a daily passive treatment consisting of: 160 μg/dose of 2KD1+3B2 200 μg/dose of 2KD1+3B2, 200 μg/dose of 2KD1 or 200 μg/dose of 3B2. Untreated animals were included as a control group. Results for diarrhea and intestinal infectious virus titers are depicted in Fig 4. At PID 2, mice from all experimental groups developed diarrhea. At PID 6, the percentage of mice with diarrhea significantly decreased in groups receiving 200 μg of 2KD1+3B2 (p = 0.0003), 200 μg of 2KD1 (p = 0.0003) and 200 μg of 3B2 (p = 0.0009) when compared to the untreated control. By PID 7, none of the mice in these groups showed any signs of diarrhea (Fig 4)showing a significant reduction in diarrhea duration from 6.75 days in the untreated group to an average of 4.75 days in mice receiving a 200 μg/dose of the VHH (p = 0.002). Overall, no significant differences were found between groups treated with 200 μg of 2KD1, 3B2 or the 1:1 mixture of both clones (p>0.05). On the other hand, mice treated with 160 μg of the VHH mixture evidenced a trend to decrease the percentage of mice affected with diarrhea from PID 6 (62.5%) but were not able to significantly reduce diarrhea duration when compared with untreated animals (p>0.05).

Regarding infectious RVA titers in intestinal samples, mice treated with 160 and 200 μg of 2KD1+3B2 and 200 μg of 2KD1 only showed infectious RVA particles at PID 1. By PID 2, these treated groups evidenced significantly lower titers when compared to the untreated control group (p = 0.02), which showed persisting intestinal RVA infection from PID 1 to 6 (Fig 4). On the other hand, mice receiving 200 μg of 3B2 showed conflicting results, as some of the litters (50%, 2/4) presented sustained RVA infection throughout the treatment, while others tested negative along the experiment (Fig 4), although RVA titters were lower than in the untreated control at all times. All treated groups, except for the one receiving 200 μg of 3B2, significantly diminished the severity of intestinal RVA infection when compared with the untreated control (AUC comparison, p = 0.04).

**Fig 5:**
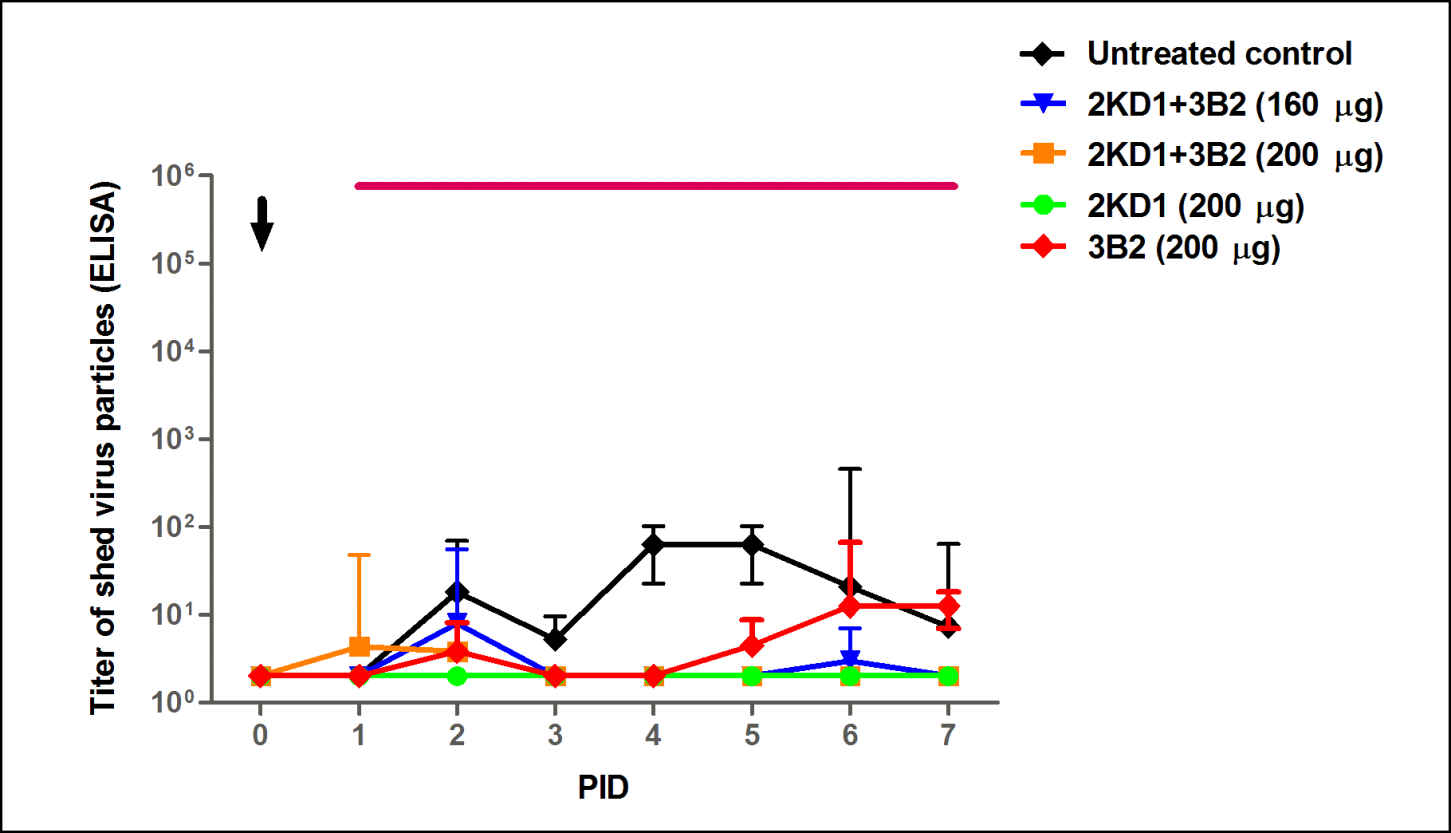
Effect of pre-symptomatic therapeutic administration of 2KD1+3B2 VHH on RVA shedding in feces. Mean titer of virus shed (determined by ELISA) detected daily in pooled feces for mice receiving pre-symptomatic therapeutic passive treatment with VHH and the untreated control group. The arrow shows virus inoculation time (PID 0) and the thin horizontal line indicates the duration of VHH passive treatment. AUC comparison showed that all VHH-treated groups, except for the ones receiving 200 μg of 3B2, significantly diminished overall fecal shedding with respect to the untreated control (ANOVA, p = 0.04). PID: Post Inoculation Day.

Therapeutic use of the VHH also achieved a significant reduction of RVA shedding in feces in all VHH-treated groups when compared to the untreated group (AUC comparison, p = 0.01), which showed continuous shedding of viral particles from PID 2 to 6 (Fig 5). Mice treated with 200 μg of 2KD1+3B2 only displayed viral shedding at PID 1 and 2 whereas mice receiving 200 μg of 3B2 showed viral shedding at PID 2 and 5–7 (Fig 5). Mice receiving 160 μg of 2KD1+3B2 presented a higher RVA shedding peak at PID 2 and a lower one at PID 6. Interestingly, mice receiving 200 μg of 2KD1 did not display detectable RVA shedding at any time (Fig 5).

At PID 8, all VHH-treated mice showed similar anti-RVA IgA Abs titers than the untreated control group in intestinal contents (p>0.05) (Fig 4). Even when IgA Ab titers rose from PID 8 to 40 in all groups, the untreated control showed significantly higher titers than all VHH treated ones at PID 40 (p = 0.03) (Fig 4). Likewise, at PID 8 all treated groups presented detectable titers of serum anti-RVA IgG Abs and no significant differences were detected with respect to the untreated control group (p>0.05). At PID 40, IgG Abs titers to RVA were significantly higher in the untreated control group than in mice treated with VHH (p = 0.002). Mice receiving 160 μg of the VHH presented similar titers at PID 40 than at PID 8.

### Post-symptomatic therapeutic treatment of RVA-associated diarrhea with anti-VP6 VHH significantly decreased the severity of intestinal RVA infection and shedding in feces

Mice received the first passive treatment dose after the occurrence of diarrhea at PID 2 (Fig 1). A group of four day-old mice -comprising three litters- was inoculated with murine ECw RVA strain (G16P[16]I7). Mice received 200 μg of 2KD1 from PID 2 to PID 7 (7-day-long treatment).

**Fig 6:**
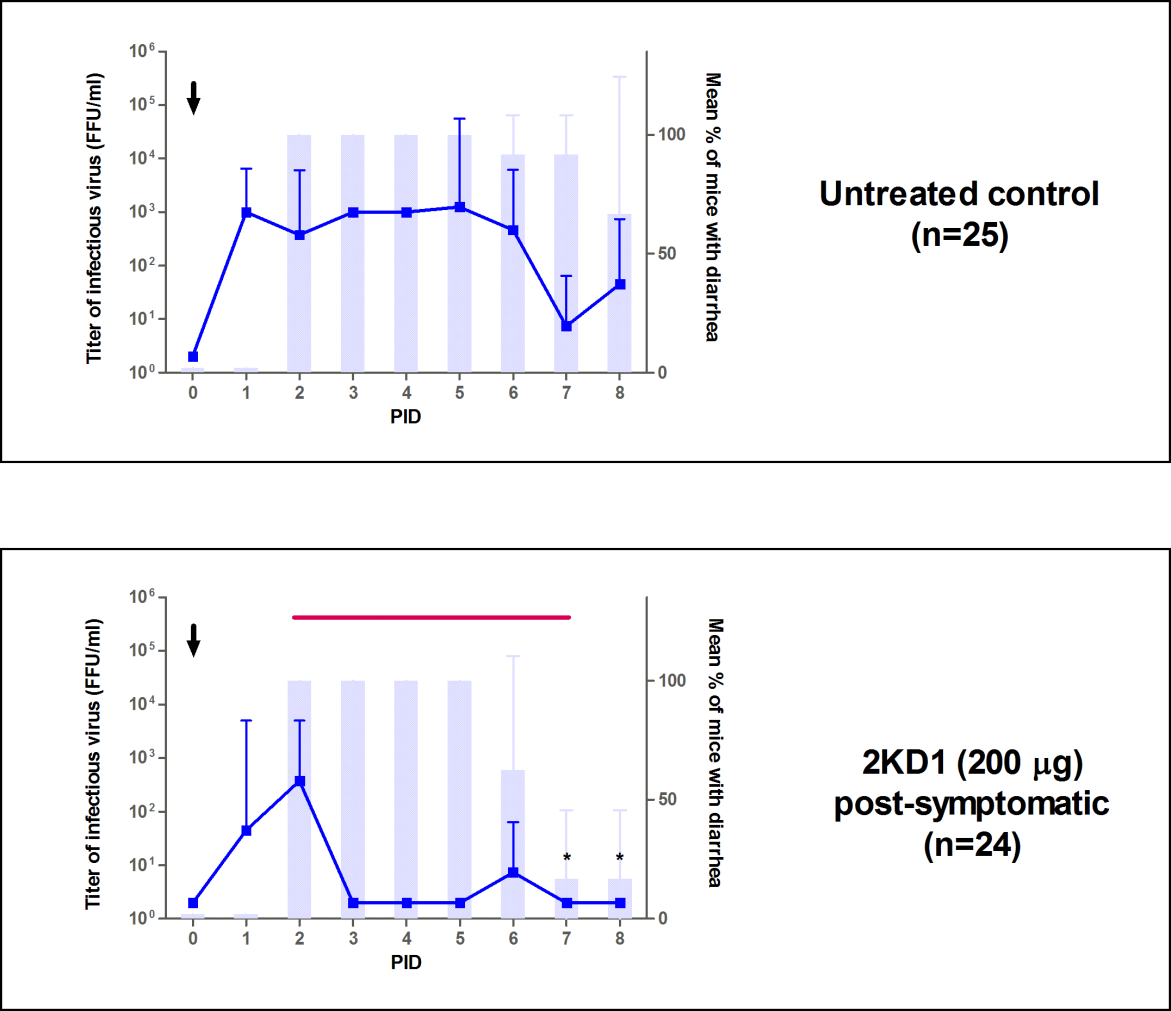
Effect of post-symptomatic therapeutic administration of VHH on RVA-associated diarrhea in neonatal mice. All mice were orally inoculated with ECw RVA at day four of life (PID 0), as indicated by the arrow. Mice were euthanized sequentially to obtain intestinal tissue (3–4 mice per time point). Blue lines depict the development of intestinal RVA disease while vertical bars show the mean percentage of mice with diarrhea per group. The * symbol indicates that the percentage of mice with diarrhea was significantly lower than in the untreated control group (Fisher Exact test). The thin horizontal line shows the duration of the passive treatment. PID: Post Inoculation Day.

As expected, by the beginning of the passive treatment, all mice had developed diarrheal symptoms (Fig 6). This situation remained until PID 6, where a marked reduction in diarrhea was observed in mice treated with 200 μg of 2KD1 compared to the untreated control (Fig 6). By PID 7, only 16.67% of treated mice presented symptoms against 91.67% in the untreated control group (p = 0.0008). Although there was a trend towards a decrease in diarrhea duration and severity in VHH-treated mice, it did not present significant differences with the untreated control group (p>0.05).

**Fig 7:**
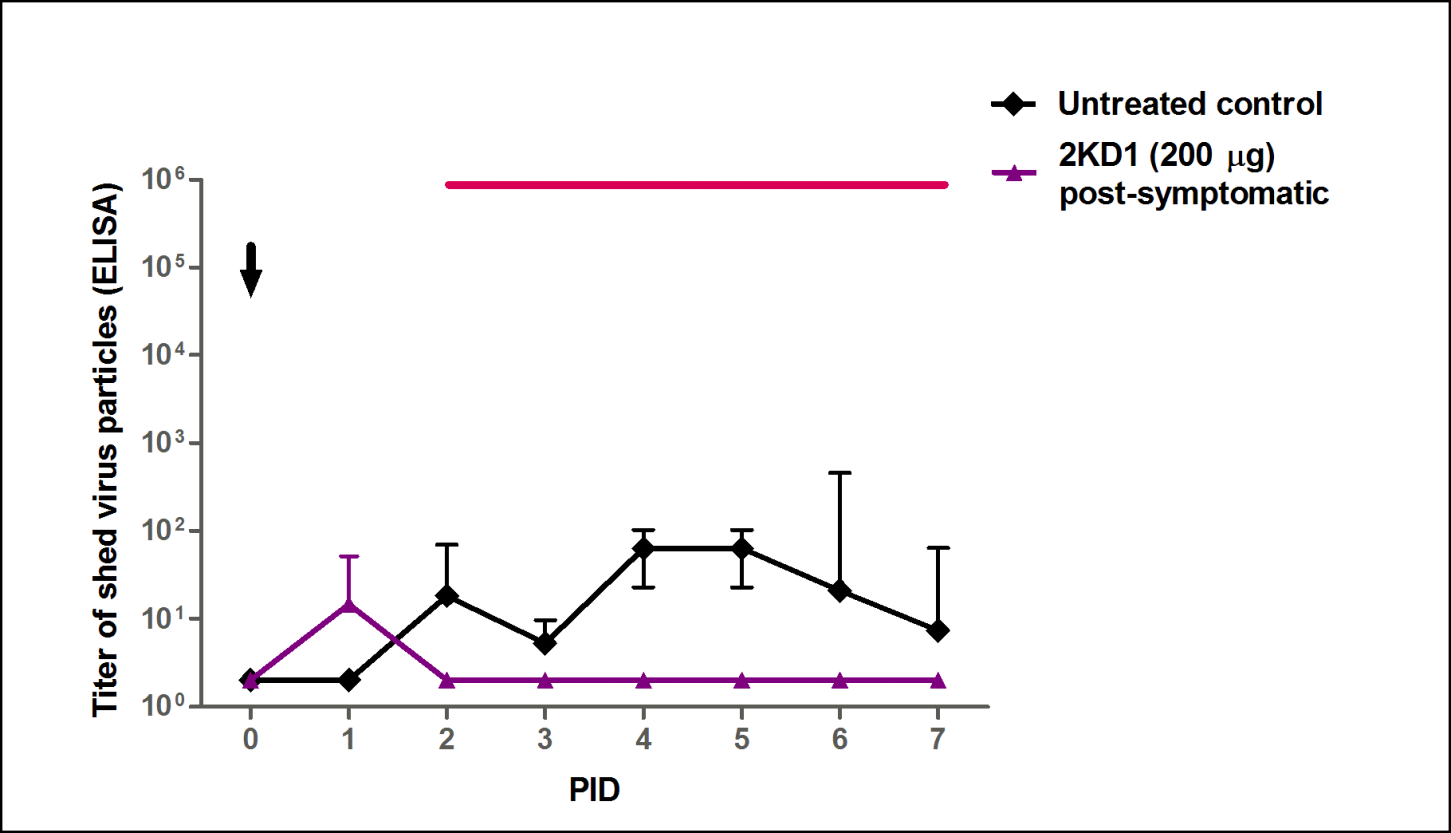
Effect of post-symptomatic therapeutic administration of anti-VP6 VHH on RVA shedding in feces. Mean titer of virus shed (determined by ELISA) detected daily in pooled feces from mice receiving pre-symptomatic and post-symptomatic treatment comprising 200 μg of 2KD1 and the untreated control group. The arrow shows inoculation time (PID 0). Post-symptomatic administration of 2KD1 (200 μg) significantly reduced overall fecal shedding with respect to the untreated control group when AUC comparison was performed (ANOVA, p = 0.002). The thin horizontal line indicates the duration of VHH passive treatment. PID: Post Inoculation Day.

Infectious RVA particles in the intestinal contents were first detected at PID 1 in both groups (Fig 6). However, mice treated with 200 μg of 2KD1 showed significantly lower viral titers than the untreated control group by PID 3 (p = 0.006; Fig 6). While untreated mice displayed persisting RVA titers until PID 7, animals receiving the VHH passive treatment showed detectable viral titers only at PID 1–2 and 6. Post-symptomatically treated mice also achieved a significant reduction of RVA shedding in feces when compared to the untreated control group (AUC comparison, p = 0.002), presenting detectable viral shedding only at PID 1–2 (Fig 7).

### Host specific humoral immune response against VHH was undetectable up to 60 days post inoculation

**Fig 8:**
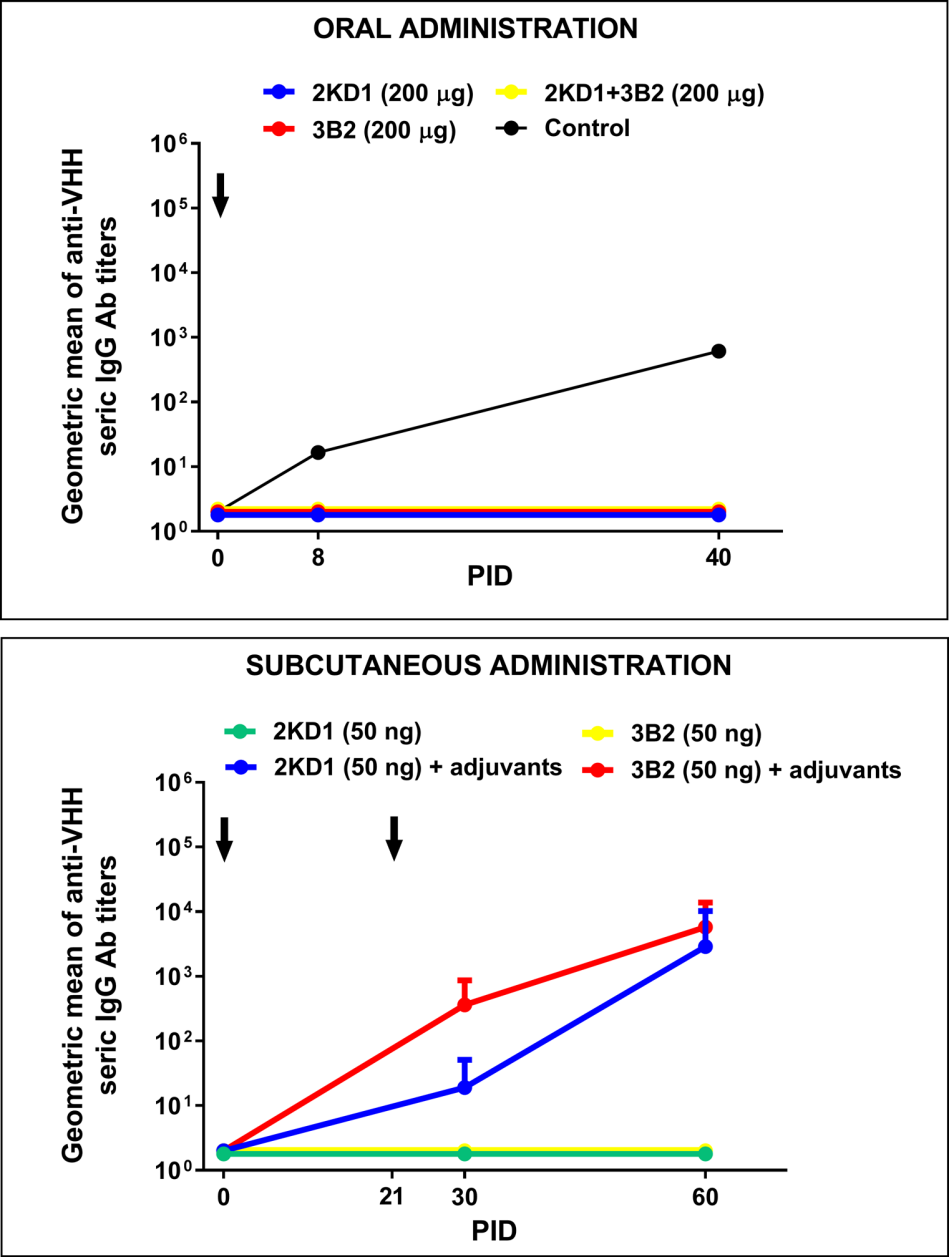
Humoral Ab responses against anti-RVA VHH in mice treated with anti-VP6 VHH. The panel above shows seric IgG Ab response against anti-VP6 VHH in neonatal mice that received oral therapeutic treatment for seven days, consisting of: 2KD1 (200 μg), 3B2 (200 μg) and 2KD1+3B2 (200 μg). The control employed for this assay was serum from an adult mice immunized subcutaneously with 2KD1 (50 ng) in the presence of adjuvants. The panel below shows seric IgG Ab response against 2KD1/3B2 in adult mice immunized subcutaneously with 50 ng of VHH at PID 0 and 21 with or without adjuvants. For this experiment, four adult mice per group were used. The adjuvant employed was Freund’s complete adjuvant for the first immunization, while Freund´s incomplete adjuvant was used in the second one. Arrows indicates immunization time for both experiments.

Murine anti-RVA VHH were not detected in treated mice serum throughout the experiment (data not shown). Anti-VHH IgG (from serum samples) and IgA (from intestinal contents) Abs were measured at PID 0, 8 and 40 against 2KD1 and 3B2 in separated assays using samples from mice treated therapeutically with the highest dose (200 μg) of 2KD1, 3B2 and 2KD1+3B2. None of the treated mice presented detectable IgG Abs against VHH in serum even after seven days of continued administration and up to PID 40 (Fig 8). We could not detect intestinal IgA Abs against the VHH, suggesting that a local humoral immune response was not developed either (data not shown). Regarding experiments in which adult mice were immunized subcutaneously with 2KD1 or 3B2, we found that mice immunized in the presence of adjuvants showed high titers of seric IgG Abs to the VHH at PID 60 (Fig 8). On the other hand, mice immunized only with VHH without adjuvants did not develop an immune response against the VHH, showing no detectable Ab titers against 2KD1/3B2 up to PID 60 (Fig 8).

### RVA escape mutants to the anti-VP6 VHH treatment could not be detected *in vivo*

**Fig 9:**
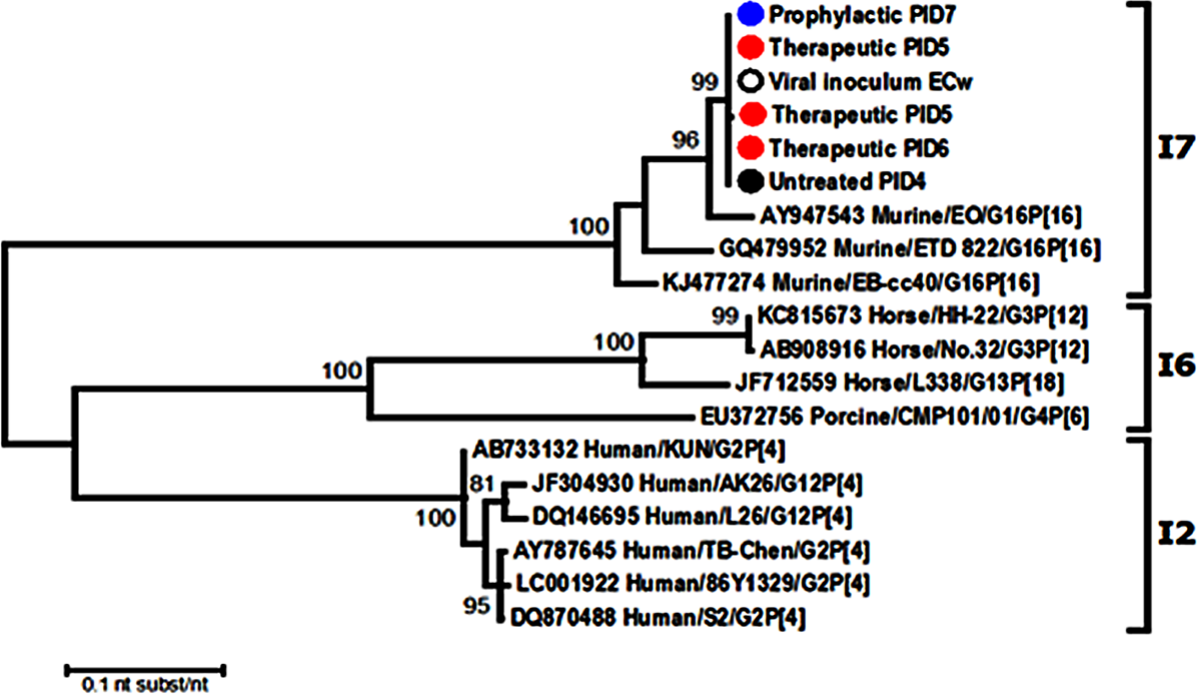
Phylogenetic analysis of VP6 sequences. The phylogenetic tree was constructed using the Maximum Likelihood method based on the Tamura 3-parameter model. Bootstrap statistical support (1000 repetitions) is shown. All the used strains are named with the Genbank accession number and strain name. The experimental samples are indicates as: blue circle = Prophylactic PID 7; red circle = therapeutic PIDs; black circle = untreated and; white circle = viral inoculum.

In the prophylactic and therapeutic assays conducted in this work we found intestinal and fecal samples from VHH-treated mice that tested positive for RVA at PID 5–6 (Fig 2 and Fig 4). Given that previous samples from these groups were negative for RVA, we aimed to elucidate if these RVA-positive samples may represent VP6-escape mutants to the passive treatment. A set of four samples of VHH-treated mice from different groups were further studied: one sample from mice treated prophylactically with 160 μg of 2KD1+3B2 (Fig 2) and three samples from mice treated therapeutically with 200 μg of 3B2 (Fig 4). We also included one sample from untreated control group and the viral inoculum as negative controls. Analyses of the obtained VP6 gene sequences showed no nucleotide differences among samples from VHH-treated and untreated mice (100% nucleotide sequence identity for complete VP6 coding sequence) (data available upon request). The phylogenetic analysis was consistent and our samples represent a unique branch that included the viral inoculum, untreated, prophylactic and therapeutic samples (Fig 9). Finally these samples tested positive by CCIF for RVA using both VHH, 3B2 and 2KD1, labeled with Alexa fluor.

### Anti-RVA VHH 3B2 and 2KD1 resisted gastric enzymes in the presence of ORS

**Fig 10:**
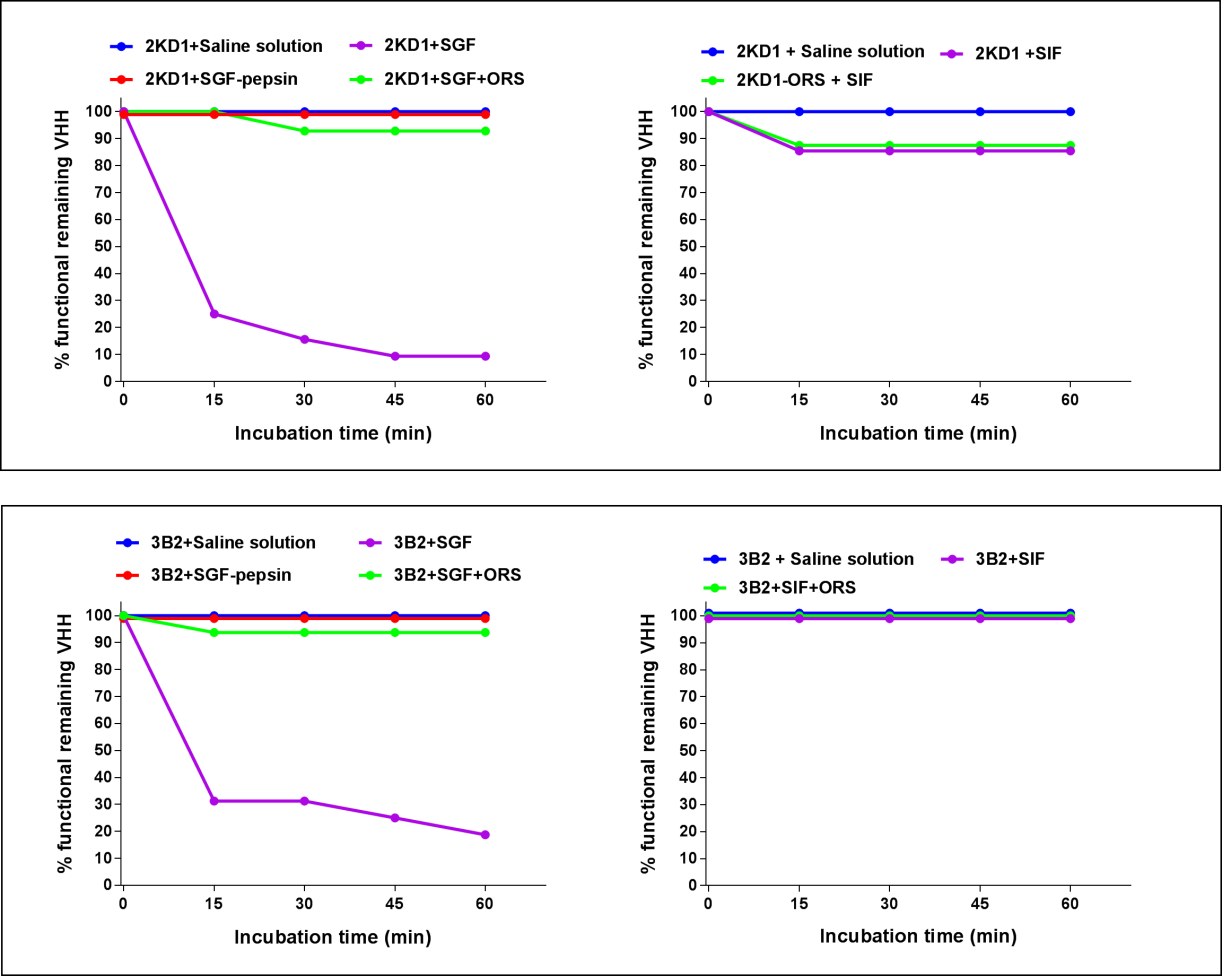
Ph stability and proteolytic resistance of anti-VP6 VHH. The graphs show the percentage of remaining functional 2KD1/3B2 after incubation in SGF (pepsin 0.32% w/v; pH 1.2) or SIF (pancreatin 1%, w/v; pH 6.8) in different conditions, determined by RVA VHH Ab titer ELISA. The panel above shows pH stability and proteolytic for 2KD1 clone whereas the panel below depicts these parameters for 3B2. In all cases 400 μg of the VHH were incubated for one hour at 37°C. Experiments were conducted by triplicate. SGF: Simulated Gastric Fluid. SIF: Simulated Intestinal Fluid. ORS: Oral Rehydration Salts.

Regarding proteolytic resistance to pepsin present in SGF, both 2KD1 and 3B2 were shown to be severely degraded by gastric pepsin, with less than 10% of 2KD1 and less than 20% of 3B2 remaining functional after incubation for one hour (Fig 10). On the other hand, we could determine that the VHH presented high stability at pH 1.2 and that the observed degradation was only caused by pepsin activity and not by low pH conditions. Interestingly, when both clones were incubated in SGF in the presence of ORS degradation by pepsin was prevented, with over 90% of the VHH remaining functional after one hour incubation (Fig 10). On the other hand, 2KD1 and 3B2 samples incubated with SIF did not show any significant sign of degradation with around 95% of the VHH remaining functional after one hour incubation (Fig 10). These results suggest that the critical step for an efficient intestinal delivery of the VHH would be achieving resistance to gastric pepsin.

### 2KD1 showed higher binding affinity to RVA particles than 3B2

**Fig 11:**
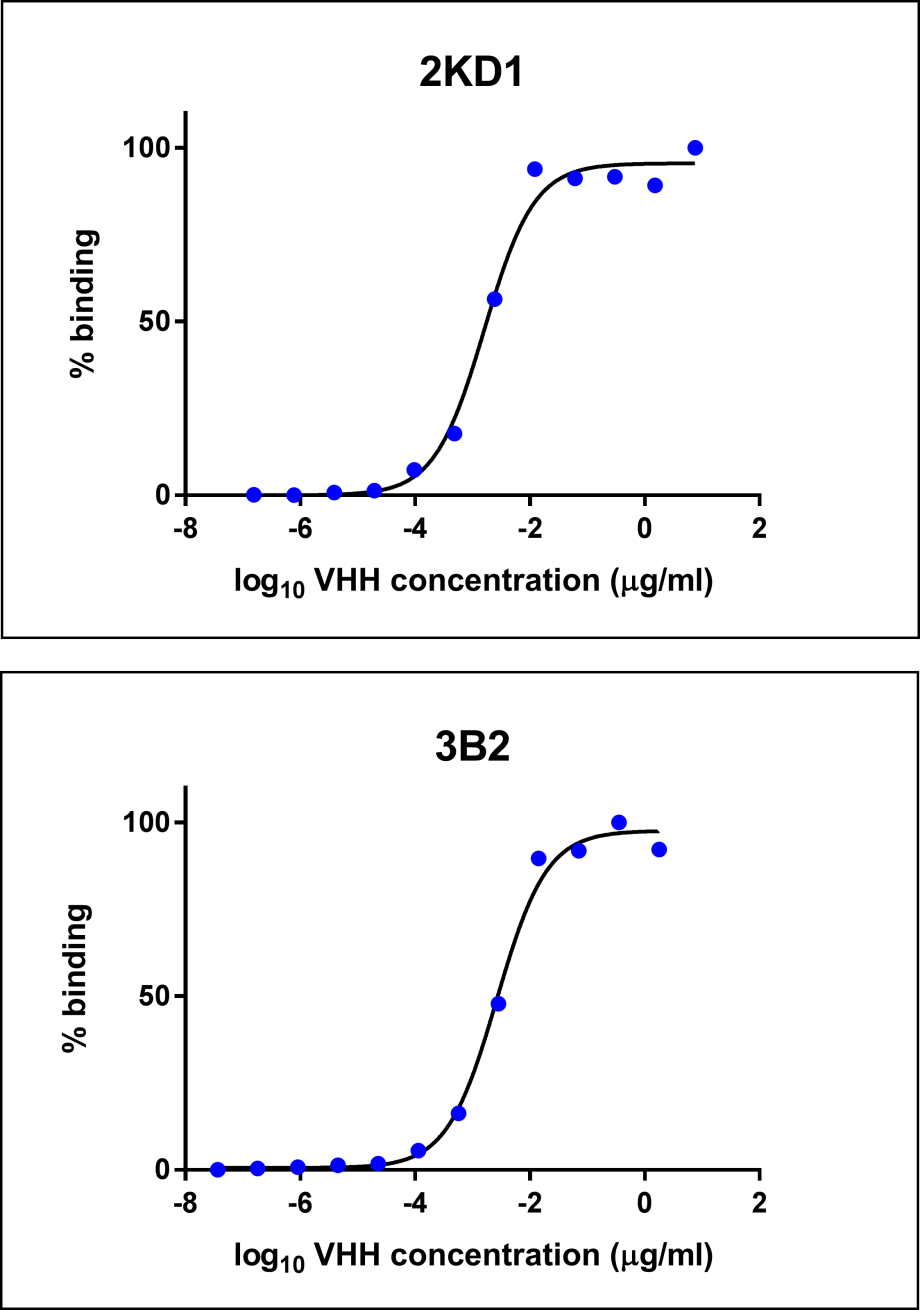
Binding affinity of anti-VP6 VHH to RVA particles. Decreasing concentrations of both 2KD1 and 3B2 were tested for their binding affinity to immobilized RVA particles in an ELISA previously described. Dose-response curves were fitted using the dose-response sigmoidal model in GraphPad 7 software (available online) obtaining R square values of 0.990 for 2KD1 and 0.991 for 3B2. The EC50 values were calculated for both clones, showing that 2KD1 had a three-fold higher binding affinity for RVA particles than 3B2: 0.0016 μg/ml vs 0.0048 μg/ml.

The binding affinity to the viral target was three-fold higher for 2KD1 than for 3B2, with EC50 values of 0.0016 μg/ml and 0.0048 μg/ml respectively (Fig 11). Both VHH clones were able to neutralize a broad range of RVA strains (S1 Table) including worldwide prevalent human RVA strains such as Wa (G1P[8]I1) and DS1 (G2P[4]I2) and local strain Arg720 (G12P[9]I?). For some RVA strains, including the murine RVA strain used in mice experiments, 2KD1 seemed to be more potent than 3B2, as up to four-fold lower concentrations of 2KD1 achieved successful neutralization of viral particles (S1 Table).

## Discussion

RVA gastroenteritis remains a major cause of infant morbidity and mortality with 90% of RVA-associated deaths occurring in low-income settings [59–61]. Previous studies have shown that administration of anti-RVA VHH mitigated or prevented the occurrence of RVA-induced diarrhea in the neonatal mouse and the gnotobiotic pig model of RVA infection and disease, respectively. Anti-RVA VHH were successfully produced in genetically engineered probiotics (*L*. *paracasei*) [46–48] which represents an affordable and efficient treatment strategy that could minimize concerns about the use of Abs within affected children’s households given current widespread of probiotics. Another interesting approach was the expression of anti-RVA VHH in transgenic rice plants, given that it combined the astringent properties of rice water against diarrhea together with specific anti-RVA Abs [43]. However, these studies focused only on the preventive use of VHH against RVA-induced diarrhea.

The main goal of this work was to evaluate the use of two clones of anti-VP6 VHH Abs -2KD1 and 3B2- in the therapeutic treatment of RVA-induced diarrhea using a neonatal mouse model infected with a high dose of murine RVA (1778 DD_50_ of EcW strain). Although neonatal mice are also susceptible to other strains of RVA until 14 days of life, we chose to use an homologous RVA because it is more virulent and replicates more efficiently in mouse intestine than heterologous RVA strains [62]. It has been demonstrated in suckling mice that enteric replication of murine RVA ECw is 1000 to 10,000-fold greater than that of a simian RVA strain [62]. Previous studies that evaluated other VHH (ARP1) for the treatment of RVA-induced diarrhea employed a neonatal mouse model infected with simian RVA (Rhesus strain, RRV). More recently, a field trial involving pediatric patients showed that, while ARP1 VHH successfully decreased total stool output and rehydration requirements, it failed to reduce the duration of diarrhea [49], when previous assays in neonatal mice infected with Rhesus RVA had decreased diarrhea duration among treated animals [44,50]. Despite obvious limitations for extrapolating results from animal models to field trials involving human patients, these differences among RVA strains could suggest that the neonatal mouse model infected with a homologous strain could predict more accurately the efficiency of an anti-RVA treatment in human patients [63].

The prophylactic administration of VHH Abs was studied to further understand this approach in a neonatal mice model followed daily further than PID 4 using a mixture of two anti-RVA VHH clones (2KD1 and 3B2) to reduce the possibility of emergence of RVA escape mutants to the treatment. A reduction in the duration of RVA diarrhea that was achieved by VHH represents the most desirable outcome of any treatment or vaccination, given that even anti-RVA vaccines do not prevent subsequent infections but mitigate the severity and duration of the symptoms. Reduction of fecal RVA shedding to undetectable levels is a key feature in preventing RVA dissemination to naïve individuals. Humoral immune responses to RVA were detected in all inoculated mice, despite the administration of passive prophylactic treatments, which are critical to prevent RVA diarrhea after a second exposure to the virus [64,65]. Overall, the prophylactic administration of anti-VP6 VHHs represents an effective prophylactic strategy against RVA-associated disease and could be used as a complement tool to RVA vaccines in populations with reduced immunization efficacy.

Post-inoculation treatment with all VHH combinations (2KD1, 3B2, 2KD1+3B2) achieved a reduction in the duration of diarrhea (in two days) and the severity of the disease. This represents a promising outcome for a therapeutic treatment against RVA-induced diarrhea administered after viral inoculation. The observed persistence of diarrhea after viral clearance could be explained by the virulence of the murine RVA strain (ECw) used for inoculation. Early studies using the neonatal mouse model for murine RVA infection reported that once RVA was undetectable at the tissue level, repair of the absorptive cell layers sufficient to restore the normal transport of fluids across the epithelium facing the gut lumen required more time [66]. It has been suggested that RVA-associated diarrhea entails a secretory component mediated by Enteric Nervous System activation and by viral NSP4 [67]. This non-structural protein pays a role in the regulation of cellular calcium homeostasis and acts as an enterotoxin [67].

Interestingly, the same dose of 3B2 (200 μg) was not able to significantly diminish the severity of RVA infection in intestinal tissue and showed persisting lower infection titers throughout the experiment. This may indicate that the administration of 2KD1 alone represents the treatment of choice given that it was also capable of reducing RVA shedding in feces to undetectable levels. These findings are in accordance with previous studies showing that 2KD1 had a deeper impact on fecal shedding than 3B2 [37] and with binding affinity experiments conducted in this work. Clone 2KD1 showed a three-fold higher binding affinity (EC50) to RVA particles than 3B2 which could explain, at least partly, the higher efficacy in treating RVA-induced diarrhea. Also, 2KD1 was able to neutralize some RVA strains more effectively than 3B2, including murine RVA ECw strain used in mice experiments, which could also explain the better performance of 2KD1 observed in this study. Future studies will focus on determining the exact epitopes of VP6 to which 2KD1 and 3B2 bind and the affinity of these attachments using Surface Plasmon Resonance (SPR) equipment.

Given that administration of 2KD1 (200 μg) attained the best results in the pre-symptomatic treatment of RVA-induced diarrhea, we selected this treatment for a final experiment involving a post-symptomatic approach. In this case, VHH treatment started on PID 2, at which time all mice had developed diarrhea. Post-symptomatic therapeutic administration of 2KD1 achieved a reduction in the severity of intestinal infection and fecal RVA shedding. It also showed a trend towards the reduction of the percentage of mice with diarrhea by the end of the experiment. These results show that administration of anti-VP6 VHH successfully mitigates RVA infection, even after the onset of the symptoms, a scenario that emulates more accurately the potential use of the VHH in pediatric patients.

There are two critical factors regarding any passive Ab therapy: one is the development of a host immune response to the treatment, especially for heterologous Abs, while the other is the appearance of virus escape mutants. VHH-treated mice did not develop detectable humoral Ab responses against the passive VHH treatment (in sera or intestinal samples) by the methodology used. Furthermore, we could not detect translocation of either VHH clone to peripheral blood. In addition, adult mice vaccinated subcutaneously with VHH without any adjuvants failed to develop an immune response at least up to 60 days post-immunization whereas mice vaccinated in the presence of Freund’s complete and incomplete adjuvants presented high titers of anti-VHH serum IgG. These results suggest that 2KD1 and 3B2 do not show immunogenic properties on their own for as long as 60 days post administration even when delivered by a systemic route. This supports that oral VHH administration should not represent a hazard for pediatric patients.

On the other hand, we studied candidate samples in the search of RVA escape mutants to VHH *in vivo*. However, no such mutants were detected by the methodology used. The presence of viral particles in VHH-treated mice at PID 5–6 may represent a delay in virus replication due to the passive treatment that is soon controlled by the host local Ab immune response. As mentioned above, VP6 protein is highly conserved in different RVA strains and this may explain the absence of escape mutants. However, it is important to consider that the observation period after VHH treatment was not long enough to reach any decisive conclusions on this matter. Also, the fact that the VP6 epitope to which the VHH bind has not been elucidated also hinders the detection of possible RVA escape mutants. Further studies on this matter should include structural virology analyses of VHH-RVA particles interaction, *in vitro* passages of RVA in the presence of large concentrations of VHH and deep sequencing analyses of potential viral escape mutants.

In this study, we described pre- and post-symptomatic therapeutic treatment of RVA-induced diarrhea by oral administration of anti-VP6 VHH with broad neutralizing activity against several RVA strains circulating globally in humans and animals in the neonatal mouse infected with a murine RVA strain. One of the main challenges of orally administered Abs is avoiding degradation by gastric enzymes and resisting low pH conditions. Although incubation of 2KD1 and 3B2 with SGF showed high degradation of the VHH by gastric pepsin, addition of ORS enhanced the proteolytic resistance of the VHH. Given that ORS are regularly administered to RVA-infected children, this could be an affordable and efficient delivery of the treatment in low income settings where it could even be provided as lyophilized powder which avoiding the need of maintaining cold chains. Given that RVA-associated symptoms sometimes include severe vomiting, it remains a critical aspect for an oral therapy for this disease how these symptoms could affect an efficient delivery of the treatment to the intestine. The absence of vomiting reflex in most animal models impairs to take this factor into account in pre-clinical assays.

This study represents a proof of principle of the efficacy of VHH oral therapy to treat RVA diarrhea, increasing the understanding of these valuable Abs. We have addressed the possible emergence of RVA VP6 escape mutants against VHH treatment *in vivo* (although over a short observation period) and the potential development of a host humoral immune response against the passive treatment. Overall, oral therapy using anti-VP6 VHH has enormous potential to be implemented in developing countries, where RVA mortality is still high and current vaccines seem less efficacious, and also to be administered to prematurely born or immunedeficient children worldwide.

## Supporting Information

S1 Fig SDS-PAGE stained with Coomassie Blue that shows the electrophoretic profile of VHH production. Lane 1: 2KD1 (after purification); lane 2: 2KD1 (periplasmic extract); lane 3: 2KD1 control; lane 4: 3B2 (after purification); lane 5: 3B2 (periplasmic extract); 3B2 control. Control VHH were obtained from previous productions.
(TIF)

S1 Table *In vitro* RVA fluorescent focus reduction assay using anti-VP6 VHH. A fourfold dilution of each VHH clone (2KD1, 3B2 or non-related VHH) was mixed with an equal volume of RVA containing 100 focus forming units (FFU) of each strain. The numbers represent the minimum VHH concentration that reduced 80% of the number of fluorescent focus forming units (FFU) of each RVA strain. Neg = no neutralizing activity was observed at the highest concentration tested (62.5 μg VHH/ml). Nd = not determined. ^#^ Results obtained from Garaicoechea *et al*., 2008 [37]. * Results extracted from Vega *et al*., 2013 [36]
(DOCX)
