# Supplementary figures and images for: Anti-VP6 VHH: An Experimental Treatment for Rotavirus A-Associated Disease
Source: PLoS One. 2016 Sep 7;11(9):e0162351. doi: 10.1371/journal.pone.0162351 (PMC5014449; doi:10.1371/journal.pone.0162351)

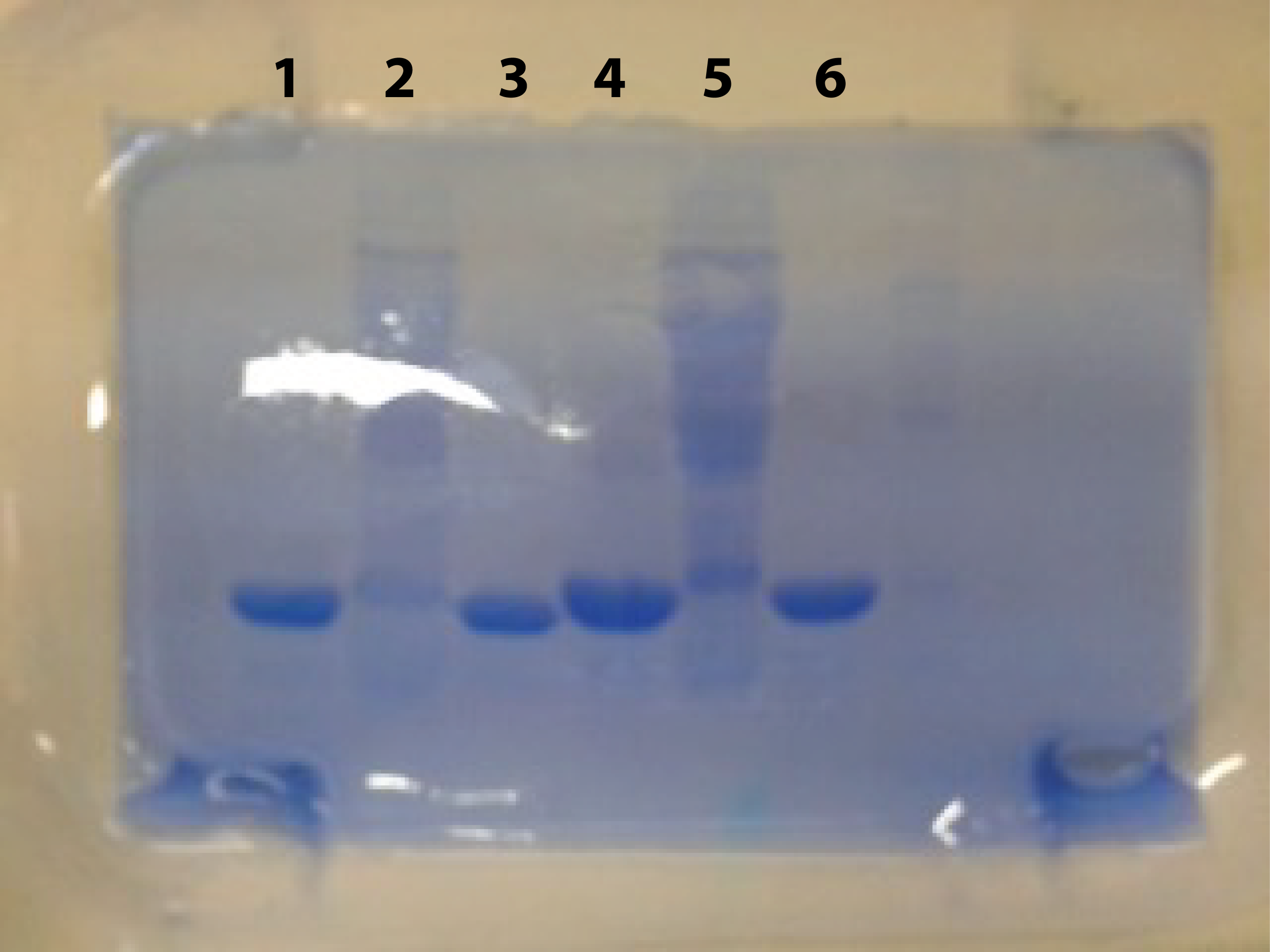

Supplement: S1 Fig — Lane 1: 2KD1 (after purification); lane 2: 2KD1 (periplasmic extract); lane 3: 2KD1 control; lane 4: 3B2 (after purification); lane 5: 3B2 (periplasmic extract); 3B2 control. Control VHH were obtained from previous productions. (TIF) [file pone.0162351.s001.tif]
