# Supplementary material for: Anti-VP6 VHH: An Experimental Treatment for Rotavirus A-Associated Disease
Source: PLoS One. 2016 Sep 7;11(9):e0162351. doi: 10.1371/journal.pone.0162351 (PMC5014449; doi:10.1371/journal.pone.0162351)
Supplement: S1 Table — A fourfold dilution of each VHH clone (2KD1, 3B2 or non-related VHH) was mixed with an equal volume of RVA containing 100 focus forming units (FFU) of each strain. The numbers represent the minimum VHH concentration that reduced 80% of the number of fluorescent focus forming units (FFU) of each RVA strain. Neg = no neutralizing activity was observed at the highest concentration tested (62.5 μg VHH/ml). Nd = not determined. # Results obtained from Garaicoechea et al., 2008 [37]. * Results extracted from Vega et al., 2013 [36] (DOCX) [file pone.0162351.s002.docx]

| **RVA strain** | **Neutralizing VHH concentration (ug VHH/ml)** | | | |
| --- | --- | --- | --- | --- |
|  | **2KD1** | **3B2** | | **Non-related VHH** |
| Wa (G1P[8]I1) | 0.20^#^ | 0.20^#^ | | Neg |
| Arg720 (G12P[9]I?) | 0,98 | 3.91* | | Neg |
| DS1 (G2P[4]I2) | 0.24 | 0.24* | | Neg |
| F45 (G9P[8]I?) | 0.06 | 0.06* | | Neg |
| ECw (G16P[16]I7) | 0.06 | 0.98 | | Neg |
| C486 (G6P[1]I2) | 0.98^#^ | 1.95^#^ | | Neg^#^ |
| Indiana (G6P[5]I2) | 0.98^#^ | 1.95^#^ | Neg^#^ | |
| B223 (G10P[11]I2) | 0.98^#^ | 0.98^#^ | Neg^#^ | |
| H1 (G5P[7]I5) | Nd | 0.98* | Neg* | |
| H2 (G3P[12]I6) | 3.91^#^ | 3.91^#^ | Neg^#^ | |
